# Chitosan Derivatives: Introducing New Functionalities with a Controlled Molecular Architecture for Innovative Materials †

**DOI:** 10.3390/polym10030342

**Published:** 2018-03-20

**Authors:** Waldo M. Argüelles-Monal, Jaime Lizardi-Mendoza, Daniel Fernández-Quiroz, Maricarmen T. Recillas-Mota, Marcelino Montiel-Herrera

**Affiliations:** 1 Centro de Investigación en Alimentación y Desarrollo, Hermosillo 83304, Sonora, Mexico; mrecillas@ciad.mx; 2 Departamento de Investigación en Física, Universidad de Sonora, Hermosillo 83000, Sonora, Mexico; daniel.fernandez@unison.mx; 3 Departamento de Medicina y Ciencias de la Salud, Universidad de Sonora, Hermosillo 83000, Sonora, Mexico; marcelino.montiel@unison.mx

**Keywords:** chitin, chitosan, derivatization, controlled functionalization, click chemistry, graft copolymer, cyclodextrin, dendrimer, ionic liquids

## Abstract

The functionalization of polymeric substances is of great interest for the development of innovative materials for advanced applications. For many decades, the functionalization of chitosan has been a convenient way to improve its properties with the aim of preparing new materials with specialized characteristics. In the present review, we summarize the latest methods for the modification and derivatization of chitin and chitosan under experimental conditions, which allow a control over the macromolecular architecture. This is because an understanding of the interdependence between chemical structure and properties is an important condition for proposing innovative materials. New advances in methods and strategies of functionalization such as the click chemistry approach, *grafting onto* copolymerization, coupling with cyclodextrins, and reactions in ionic liquids are discussed.

## 1. Introduction

**Figure 1:**
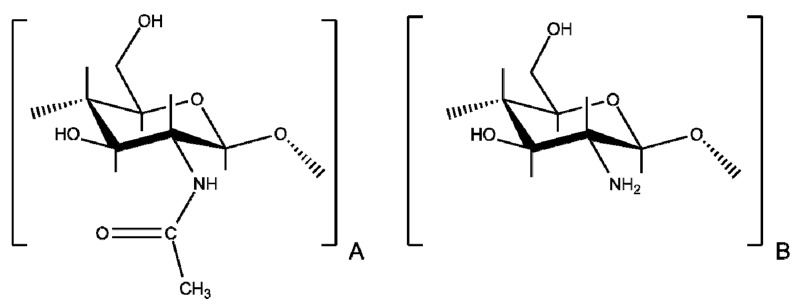
Chemical structure of chitosan composed of β(1→4) linked units of (**A**) *N*-acetyl-d-glucosamine and (**B**) d-glucosamine.

Polysaccharides are widely found in the biosphere, fulfilling various important functions in living organisms, such as energy storage and structural materials, among others. Cellulose and chitin are the most abundant natural polymers in nature. However, chitin has very few applications compared to cellulose. This is for several reasons, including the scarce natural structures of chitin that are available to be used with low processing and the poor solubility properties of this polysaccharide. Therefore, most of the obtained chitin is processed by extensive alkaline deacetylation to obtain chitosan. This amino-polysaccharide is composed of β(1→4) linked units of *N*-acetyl-d-glucosamine and d-glucosamine (Figure 1). Due to its key properties such as its biodegradability and biocompatibility, and its being mucoadhesive and non-toxic, chitosan is of great interest in many applications such as biomedicine, pharmacy, biotechnology, food industry, nanotechnology, etc. [1,2,3,4,5,6,7].

One constant topic in materials research is the modification of natural polymers, which results in the development of new derivatives with unique properties. There is a great variety of methods to modify polysaccharides. Chitosan is prone to chemical modification at free amino groups from the deacetylated units at C-2, and hydroxyl groups at C-3 and C-6 positions [2]. Commonly, the chemical derivatization of chitosan is carried out to improve some specific characteristics, such as solubility, hydrophilic character, gelling properties, and affinity toward bioactive molecules, among others [8].

Chemical modification of chitosan is usually done in bulk, giving rise to randomly reacted units. When specific new functionalities are pursued, other approaches are preferred in which the reactions can be controlled stoichiometrically. In this scenario, polymer science is taking advantage of diverse strategies to design new polymer-based hydrogels, drug and gene delivery systems, scaffolds for tissue engineering, toxic substance, mineral chelation, and materials for the electronics and aerospace industry, among others. For example, introducing lipophilic or hydrophilic molecules to chitosan may result in altering or improving its properties, such as solubility in acidic solution or organic solvents, improving thermal and mechanical properties and others [9].

There are previous reviews covering important and specific aspects of the chemical modification of chitin and chitosan [10,11,12,13,14,15,16]. In the present paper, we aim to review and analyze recent developments found in literature dealing with the chemical modification of chitin and chitosan, with emphasis on proposed methods to obtain chitosan derivatives with a controlled macromolecular architecture. An understanding of the interdependence between chemical structure and properties is an important condition for proposing innovative materials. 

Some aspects of the chemistry of these polysaccharides (and their modification conditions) could have an impact on the properties of the products and should be taken into account:

1. Chitin and chitosan are, in fact, a family of polymers, differing in terms of not only the molecular weight and extent of acetylation but also in the dispersion of the degree of polymerization and the distribution of the acetylated and deacetylated units along the polymer chain. All these parameters will depend mainly on the natural source and isolation processes. Therefore, it is very important to know these intrinsic characteristics, as far as they shall affect the properties of the derivatives.
2. Due to their insolubility in certain solvents (particularly chitin), some chemical reactions are carried out under heterogeneous conditions. This will have a determinant influence on the structure of the obtained derivatives. Using words from Kurita, “reactions under heterogeneous conditions are usually accompanied by problems including poor extents of reaction, difficulty in regioselective substitution, structurally ununiformly products, and partial degradation due to severe reaction conditions” [1]. Nowadays, these drawbacks could be circumvented using some novel solvent systems like ionic liquids. This topic will be revised herein as well.
3. Usually, non-selective chemical derivatization could lead to the development of products with an irregular distribution and uncontrolled growth of the substituent groups in the main chain, or undesired depolymerization of the polysaccharide.
4. Although chitosan presents valuable functional groups for derivatization reactions, often it is necessary to obtain some precursors to facilitate subsequent reactions or, in other cases, to protect the reactive amine in order to favor the chemoselectivity of the modification. Due to their frequent use in chitosan functionalization processes, we will first refer to those reactions whose use is more or less recurrent under diverse experimental conditions.

### 1.1. Some Frequent Reactions in Chitosan Chemistry

There is a group of organic reactions, whose use in chitin chemistry is recurrent, due to their experimental simplicity, and because their products can be used as a kind of wildcard during other chemical modification strategies. These reactions provide the researcher with valuable tools for specificity control and the regioselectivity of the functionalization, with minimal possibilities of side reactions or chain degradation. Herein, we will only make a brief summary of them, and the reader can get more details in other excellent reviews and compilations [1,10,17,18,19,20].

Among these reactions, the following will be frequently used: (i) *formation of Schiff bases (and reductive amination)*. It refers to the formation of imine products between amine and carbonylic (aldehydes and ketones) groups. This reaction is very simple and takes place under mild conditions. The imine could be easily reduced with a suitable reducing agent like sodium borohydride (or, preferably, sodium cyanoborohydride), producing selectively *N*-alkyl derivatives; (ii) *carbodiimide-mediated amidation*. There is a group of activators for the amidation of amines with carboxylic groups. When using these agents, the amidation is straightforward and usually takes place under mild conditions at room temperature. Most frequently used activators are *N*,*N*′-dicyclohexylcarbodiimide (DCC), 1-ethyl-3-(3-(dimethylamino)propyl) carbodiimide (EDC), and 4-(4,6-dimethoxy-1,3,5-triazin-2-yl)-4-methylmorpholinium chloride (DMTMM). In order to increase yields and decrease side reactions, *N*-hydroxysuccinimide (NHS) is often added.

### 1.2. Chitosan Precursors with Protected Amino Groups

Due to the high reactivity of the amino groups, these should be protected in order to encourage the functionalization reaction to take place through the hydroxyl groups. Several methods have been proposed, but until now the most frequent has been the *N*-phthaloylation of chitosan [21,22,23].

With this purpose, typically, amino groups of chitosan could be protected from unwanted reactions by means of phthalic anhydride, whose derivative, *N*-phthaloyl chitosan, protects the amine moieties for further chemical modifications. *N*-phthaloylation should be carried out in DMF/water (95/5) in order to avoid *O*-phthaloyl substitution [22]. At the end of the chemical modification, the phthaloyl protection must be removed from the polymer by reaction with hydrazine monohydrate to regenerate the free amino groups. The *N*-phthaloylation is considered one of the best ways to protect the amine moeties of chitosan; however, this strategy has two drawbacks:

- the *N*-phthaloylation of chitosan affects the solubility of chitosan in aqueous solutions. It is only soluble in aprotic polar organic solvents, which have been attributed to an increase in the crystallinity of the derivative as compared with the pristine chitosan [22,23]. Obviously, the solubility of the precursor in some organic solvents could be advantageous when the *O*-substitution reaction needs to be carried out in the latter.
- the unblocking reaction with hydrazine monohydrate severely depolymerizes chitosan chain, with the consequent weakening of its mechanical properties [24,25,26].

Another approach for protecting amines is the formation of Schiff bases with aldehydes. It has been found that *N*-aryl aldehydes are the best option, because their Schiff bases seem to disrupt interchain packing, making hydroxyl groups more accessible for reactions [17,27]. Among them, *N*-salicylidene, *N*-*m*-toluylidene, and *N*-benzylidene chitosan usually give better results [27,28,29,30,31,32]. The deprotection is easily carried out at acidic pH values, giving *O*-substituted chitosan derivatives with almost no chain degradation. Nevertheless, under certain conditions, the protection of the amine is far from complete, which is the main disadvantage of this strategy [33].

Dissolving chitosan in methanesulfonic acid has also been used for the protection of amino groups [34,35,36,37]. As it should be expected, there is an important degradation of chitosan polymer chain due to the strong acidic conditions used to dissolve chitosan [34,35].

## 2. Click Chemistry Reactions

Among the different approaches developed to produce new chitin and chitosan derivatives, the polymer scientist should take into account the way to reduce cost, time consumption during the experiment, undesired byproducts of reactions, and reduce the possible pollution to a minimum. Click chemistry is an expression coined by Sharpless for “a set of near-perfect” reactions [38], in which two functional groups exclusively react with each other. They are quick reactions and exhibit high yields; they are carried out under mild temperature conditions (25–37 °C), in a wide range of hydrogen potential (pH 4–12); they are insensitive to water and oxygen. They must generate highly regioselective products that need no complicated purification processes. An important characteristic is that they are modular reactions resembling biochemical processes in nature. Among these reactions are the following [38,39]:

- cycloaddition reactions, including those from the 1,3-dipolar family (like Huisgen reaction), and hetero-Diels-Alder reactions,
- nucleophilic ring-opening reactions in strained heterocyclic electrophiles,
- carbonyl chemistry of the non-aldol type, and
- additions to carbon-carbon multiple bonds.

**Figure 2:**
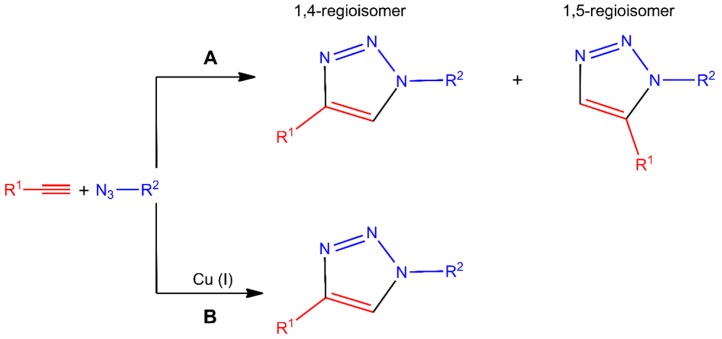
Huisgen cycloaddition reactions in the absence (**A**) or presence (**B**) of Cu(I) catalyst.

**Figure 3:**
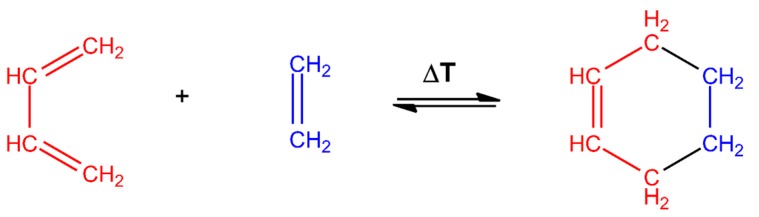
Diels-Alder reaction between a diene and a dienophile.

Among the reactions that are considered as click chemistry, cycloadditions are the most used reactions in chitosan derivatization. On the one hand, the Huisgen’s reaction is a cycloaddition between alkynes and azides yielding two regioisomer triazoles [40]. It could be carried out with or without Cu(I) as a catalyst, as could be appreciated in Figure 2. This is one of the most investigated chemoselective “click” reactions, which takes place in aqueous medium at room temperature, and is almost instantaneous. On the other hand, the Diels-Alder is a [4 + 2] cycloaddition between a diene and a dienophile, producing products with an unsaturated six-membered ring (Figure 3). This is also an important click reaction, with the peculiarity that it can be thermodynamically reversible, depending on the reactants, but its reaction product is absolutely stable [41,42,43].

The use of “click chemistry” has expanded the possibilities of producing new materials with outstanding properties [44,45,46,47,48]. Chitosan derivatives synthesized by click chemistry have shown tunable thermosensitive characteristics, photochromic behaviors, pH-sensitivity macromolecular networks, and highly soluble chemoselective properties [49,50,51,52].

The main application of click chemistry on chitosan derivatization seems to be in the preparation of grafting copolymers. Due to the chemoselectivity of these reactions, it was possible to obtain *N*- [53,54] or *O*-grafted [55,56] chitosan-*g*-poly(ethylene glycol). Other homopolymers grafted onto the chitosan backbone are poly(*N*-isopropyl acrylamide) [57,58], β-cyclodextrin (on O-6 position [31] or in the amine [59]), poly(caprolactone) [60,61], and others [62,63]. 

An interesting example of what could be prepared with this powerful tool is the study of Jung et al. in which chitosan-poly(ethylene glycol) hybrid hydrogel microparticles were prepared and then conjugated with single-stranded DNAs via Cu-free click chemistry [64]. The authors consider this strategy to be an example of a robust biomolecular assembly platform that could be replicated as a biomolecular target and in therapeutic applications.

Furthermore, there are other chitosan derivatives developed via click chemistry reactions [32,50,65,66,67,68,69,70,71,72,73,74], some of which exhibit diverse properties like antimicrobial, antifungal, enhanced solubility in acidic and basic conditions, and an antigen detection system initiated by click chemistry, etc. Other materials synthesized are a cellulose-click-chitosan material [75], click-coupled graphene sheet with chitosan [76], and chitosan functionalized multiwalled carbon nanotubes [77].

**Figure 4:**
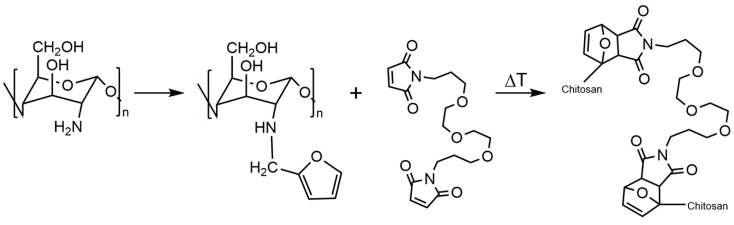
Synthetic scheme for the preparation of *N*-(furfural) chitosan by Schiff base formation/reductive amination process and Diels–Alder cycloaddition with a bismaleimide giving chitosan hydrogel.

The use of the Diels-Alder cycloaddition gives an advantage to design materials that can vary their physicochemical properties with temperature, a characteristic that Huisgen’s cycloaddition does not possess. In this sense, it could be very relevant to combine the properties of chitosan with the potential capacity of furans to carry out Diels-Alder reactions. The structure of the furan gives it a markedly dienic character, which is very suitable for the development of this type of reaction [78]. This feature opens up opportunities to investigate the potentialities of the Diels-Alder cycloaddition between furan-chitosan derivatives and dienophiles such as maleimides. This approach has been used to obtain a novel chitosan hydrogel with interesting drug-carrier characteristics that are suitable for the development of novel biotechnological and biomedical materials (Figure 4) [79].

The number of reports about the use of click chemistry to modify chitosan is growing fast. However, the application of click chemistry to the generation of regioselectively controlled chitosan derivatives is only in its early stages, and we should expect its use to have an even greater momentum in the coming years. This is undoubtedly due to the simplicity of the reactions and especially to the excellent opportunities offered by these tools to introduce chemical modifications with a high control of the molecular architecture.

## 3. Graft Copolymerization

Among the strategies available to produce chitin and chitosan derivatives, grafting procedures are a strong chemical tool that is used to develop innovative materials [11]. The structure of a typical *graft*-copolymer consists of a long sequence of the backbone polymer chain (chitin or chitosan in this case), containing one or more side polymer chain of distinct chemical nature [80]. The properties of this kind of copolymer are widely dependent on the molecular characteristics of the grafted side chains, such as molecular structure, length of the chain, and the degree of grafting [81].

**Figure 5:**
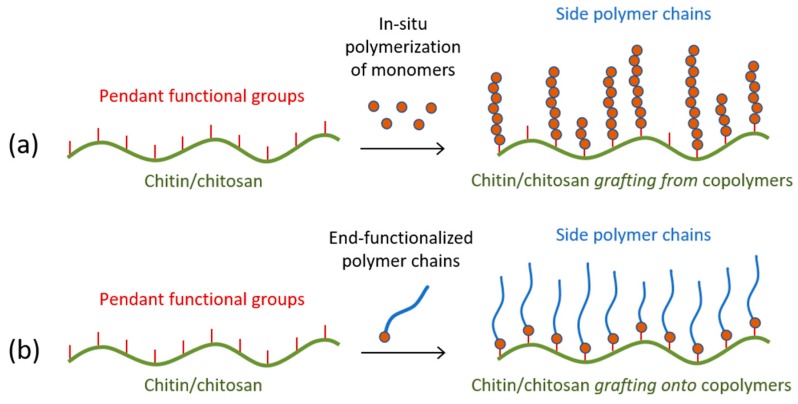
Schematic representation of the (**a**) *grafting from* and (**b**) *grafting onto* methods for graft copolymerization.

There are three main techniques for the grafting copolymerization: *grafting from*, *grafting onto*, and *grafting through*. As far as in this case the polysaccharide backbone chain is already formed, only the two former methods are of interest. On the one hand, the *grafting from* method involves the in situ polymerization of the grafting monomer (Figure 5a). This reaction is initiated directly from the main chain, but its free homopolymerization could not be discarded as well. This procedure is usually accomplished by one-step, but no control over the macromolecular structure is possible. On the other hand, the *grafting onto* method is carried through the reaction between pendant functional groups of the backbone chain and end-functional groups of previously synthesized polymer chains (Figure 5b) [80]. This procedure allows the elaboration of polymer systems with a well-defined structure. This technique affords the preparation of versatile macromolecular materials from chitin and chitosan, allowing the development of novel hybrid materials with specific properties for advanced applications in several fields as food processing, biotechnology, water treatment, biomedicine, among others.

### 3.1. Chitin “Grafting from” Copolymers

The type of polymerization to be selected depends on the type of monomer to be grafted; in most cases, radical polymerization has been used [82,83,84,85,86,87,88,89], although there is also a report of anionic ring-opening polymerization [90]. Acrylic monomers (especially acrylic acid) are among the most frequently grafted into chitin [82,83,84,85,88,91,92,93]. For the development of experimental procedures, it must be taken into account that chitin is not soluble in aqueous media and, therefore, the reaction must be carried out mostly under heterogeneous conditions. Hence, almost no-control over the macromolecular structure is attained, giving rise to a heterogeneous distribution of the grafting chains along the chitin backbone, and, in some cases, only low degrees of grafting could be reached.

The *grafting from* copolymerization of acrylic monomers onto chitin using cerium (IV) as redox initiator has been the subject of some studies [82,83,84,93]. In the pioneering work by Kurita et al., the influence of several conditions of the copolymerization reaction of acrylamide and acrylic acid onto chitin was investigated [82]. These authors reported a procedure that allows to reach the percentages of grafts above 200%. The obtained copolymers showed enhanced solubility and hygroscopic behavior [82]. Methyl acrylate [83] and methyl methacrylate [84] are other acrylic monomers that have been grafted on chitin in the past under similar conditions.

The other free radical initiator that has been successfully used for the grafting of acrylic monomers onto chitin is potassium persulfate [85,88,91,92]. Hydrogels prepared with chitin-*g*-poly(acrylic acid) by the *grafting from* method have been proposed as a wound dressing material [85,91,92]. The highly water-absorbable film showed a good capacity of absorbing exudates from wounds, thus keeping a moist wound environment [85]. Subsequently, it was shown that the inclusion of glycidyltrimethylammonium chloride improves the wound healing properties of this hydrogel [91,92].

Acrylic acid was also grafted on chitin nanofibers by the *grafting from* method using potassium persulfate. This material showed a stable dispersion in aqueous media at alkaline pH due to the stabilizing effect of electrostatic repulsions between nanofibers [88].

Chitin-*g*-polystyrene copolymer has also been prepared by the *grafting from* method using ammonium persulfate. The effect of some experimental parameters was evaluated, and the resulted material was a copolymer grafted at the C-6 position of chitin backbone [89].

Recently, polypyrrole, an electrically conducting polymer, was grafted on chitin to enhance its mechanical properties. The copolymerization reaction was carried out by the *grafting from* method using ammonium persulfate. The crystallinity of the graft copolymers decreased as a function of the increment of grafting percentage [94]. Itaconic acid, indole, and ε-caprolactone are also other examples of monomers that have been grafted into chitin by the *grafting from* procedure [86,87,90].

### 3.2. Chitosan “Grafting from” Copolymers

Unlike chitin, the copolymerization reaction of chitosan could be accomplished by the *grafting from* procedure under homogeneous conditions in aqueous media. To some degree, it allows having more control over the macromolecular structure of the obtained copolymer as compared with chitin. 

In this case, a greater variety of monomers have been grafted to chitosan via the *grafted from* procedure, for example, acrylic monomers [95,96,97,98,99,100,101,102,103,104,105,106,107,108,109,110,111,112,113], styrene [105], oligoethylene glycol methacrylate [114], *N*-vinyl-2-pyrrolidone [115,116], ε-caprolactone [36,37,116,117,118], lactide [119], urethane [120], indole [87], and aniline [121], among others. The types of polymerization and initiator to be employed depend on the selected monomer.

One of the problems of the *grafting from* procedure is the difficulty of effectively controlling the chemoselectivity of the reaction. To overcome this drawback, the *protection-graft-deprotection* method has been employed [98,116,117,118,119]. With this purpose, chitosan amino groups are initially protected by *N*-phthaloylation [22]. Then, the copolymerization reaction is conducted with *N*-phthaloyl chitosan, and, finally, amino groups are regenerated with hydrazine monohydrate. Thus, the side chains are anchored at the C-3 and C-6 hydroxyl groups of chitosan backbone, while amino groups remain free. The main disadvantage of this technique is that the copolymerization reaction should be carried out in organic solvents.

*N*-isopropyl acrylamide (NIPAm) is one of the most frequently grafted acrylic monomers on chitosan backbone, perhaps due to its thermosensitive properties and its promising applications for the preparation of advanced materials, especially on the biomedical field including drug delivery systems and tissue engineering [96,97,98,99,100,101,102,106,107,108,113]. Ammonium and potassium persulfate is the preferred radical initiator [97,99,100,102,106,107,108], but also cerium ammonium nitrate [96,101] and azo compounds [113] have been utilized. In general, the *grafting from* synthesis of poly(NIPAm) copolymers are simple and could be accomplished in one step. A strategy proposed by Chen et al. involves the synthesis via atom-transfer radical polymerization from the bromo isobutyryl-terminated chitosan at the C-6 hydroxyl group [98].

The thermosensitive properties of PNIPAm are governed by the variation of hydrophilic and hydrophobic interactions by increasing the temperature. At low temperatures, water molecules form regular ice-like structures around hydrophobic methyl groups. When the temperature increases, that hydrophobic hydration collapses. As a result, hydrophobic interactions are generated between the methyl groups of different segments of PNIPAm chains, giving rise to a polymer network. From a thermodynamic point of view, this phase transition should generate a loss of conformational entropy, due to the ordering of the polymer in the network, which must be compensated for by the translational entropy gain of the ejected water molecules. Therefore, as a result of the phase transition, there is an increase in the total entropy, which is greater than the enthalpy gain (the transition is endothermic), all of which results in a decrease in Gibbs free energy [13]. That is, the phase transition depends on the size and closeness of the grafted PNIPAm chain segments that are involved in the transition. Therefore, an adequate control of the molecular architecture allows one to effectively modulate the properties of the materials and their response to changes in temperature [13,96,122].

The rheological response of the solutions of chitosan-*g*-PNIPAm to changes in temperature is completely reversible. It has been postulated that the increase in the elastic response is due to the formation of hydrophobic crosslinked points at the expense of the amount of sol fraction; for that reason, “the connectivity in the gel network is governed by the net number of formed enthalpic-hydrophobic driven-junctions” [96]. The fast thermoreversible response exhibited by these copolymers could be associated with this phenomenon.

**Figure 6:**
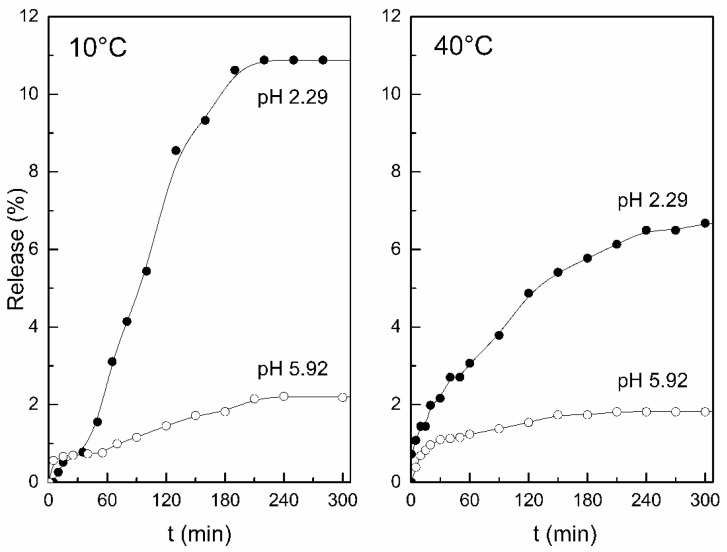
Release profile of Coomassie Blue dye from polyelectrolyte complex membranes formed between this chitosan-*g*-PNIPAm and pectin as a function of pH and temperature. Reprinted from [123], Copyright 2011, with permission from Elsevier.

Polyelectrolyte complex membranes formed between chitosan-*g*-PNIPAm and pectin exhibit temperature and pH dual-stimuli responses. Figure 6 shows the release of a model substance as a function of pH and temperature [123], and it can be appreciated how this type of material can respond simultaneously to both parameters. 

More information about the structure, properties, and potential applications of chitosan-*g*-PNIPAm copolymers could be found in other specific reviews [13,124].

ε-caprolactone is the other monomer also often grafted onto chitosan. Poly(ε-caprolactone) is a hydrophobic, biodegradable, and biocompatible polymer with excellent mechanical properties. Therefore, the search for hybrid, chitosan-based materials exhibiting the properties of both polymers is an advantageous strategy. Because one of the properties of chitosan that is important to take advantage of is its hydrophilicity and solubility in acidic aqueous solutions, the *protect-graft-deprotect* strategy has been the preferred method [116,117,118]. In this case, different synthetic approaches have been tested. The typical amino group protection by *N*-phthaloylation has been followed in most of the cases [116,117,118]. In these cases, tin octanoate was selected as a catalyst [116,117], but it has been also shown that *N*-phthaloyl chitosan is also by itself a catalyst for the ring-opening polymerization of caprolactone monomers and hydroxyl groups acting as initiators [118]. Moreover, there are other reports in which methanesulfonic acid was used as a solvent for chitosan and at the same time served to protect the amino groups, and as a catalyst for the ring-opening reaction [36,37]. This copolymer could be used as an efficient stabilizer of gold nanoparticles [116] and could form amphiphilic copolymer micelles suitable for the drug delivery of hydrophobic anticancer molecules [37].

In conclusion, it can be highlighted that the *grafting from* method has the advantage that chitosan can be functionalized in a fairly easy manner, generally in a single reaction step. If it is required to control the chemoselectivity of the copolymerization and to direct the reaction towards the hydroxyl groups at C-6 and C-3 positions, it is necessary to follow the strategy of protecting amino groups before copolymerization. The main drawback of the *grafting from* method is that there is poor control over the structure of the copolymer, both in terms of the dispersion of the grafted chain length and its distribution throughout the chitosan backbone (Figure 5a).

### 3.3. Chitosan “Grafting onto” Copolymers

As it is discussed above, the *grafting onto* is the other technique of preparing graft copolymers. Its main advantage is that it is possible to obtain derivatives with better control of the macromolecular architecture, and, therefore, it should be possible to have a greater possibility of modulating the properties and applications of these materials. There are several types of homopolymers that have been grafted onto chitosan, including poly(ε-caprolactone) [125,126,127], poly(ethylene glycol) and Pluronic [24,53,54,55,56,128,129,130,131,132,133,134,135,136,137,138,139,140,141], poly(*N*-isopropyl acrylamide) [57,58,98,142,143,144,145,146], and poly(*N*-vinylcaprolactam) (PVCL) [147,148,149,150,151,152,153,154], among others. 

**Figure 7:**
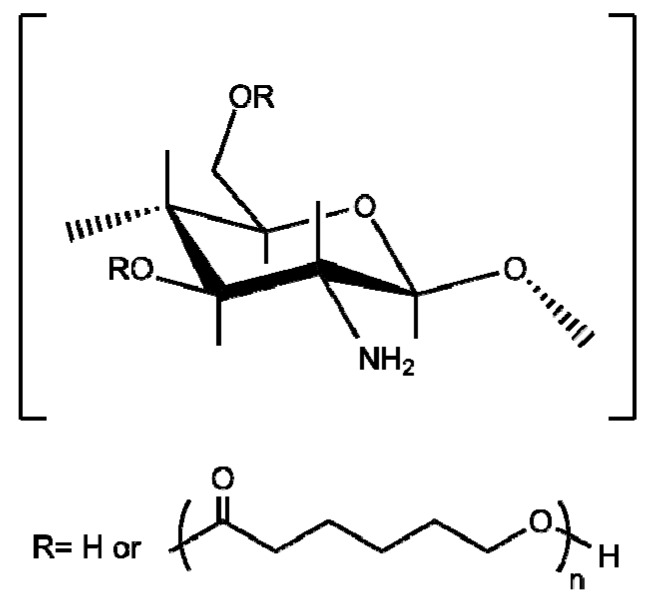
Chemical structure of chitosan-*g*-poly(ε-caprolactone).

The grafting of end-functionalized poly(ε-caprolactone) has been conducted by the *protect-graft-deprotect* procedure, using EDC condensing agent for carboxylic-terminated poly(caprolactone) (Figure 7) [126,127], or the reaction of isocyanate groups with chitosan hydroxyl groups [125]. It has been reported that the resultant material could be self-assembled into micelles and used as stabilizers to prepare silver nanoparticles with good antimicrobial activity [127].

**Figure 8:**
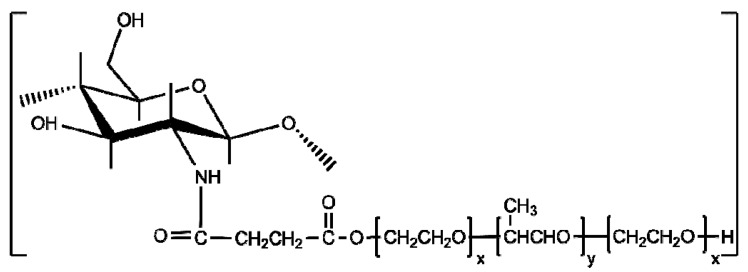
Chemical structure of chitosan-*g*-Pluronic.

Chitosan grafted with Pluronic, poly(ethylene oxide)-*b*-poly(propylene oxide)-*b*-poly(ethylene oxide), copolymer have also been synthesized by the *grafting onto* method. For this purpose, Pluronic was “activated” with succinic anhydride, and the resulted carboxylated Pluronic was grafted onto chitosan in the presence of EDC/NHS system (Figure 8) [139,140,141]. This water-soluble thermosensitive copolymer has been evaluated as a potential injectable cell delivery carrier with the aim of using it as a scaffold for cartilage regeneration [140]. Its suitability in the preparation of nanocapsules for drug delivery was also verified [141].

**Figure 9:**
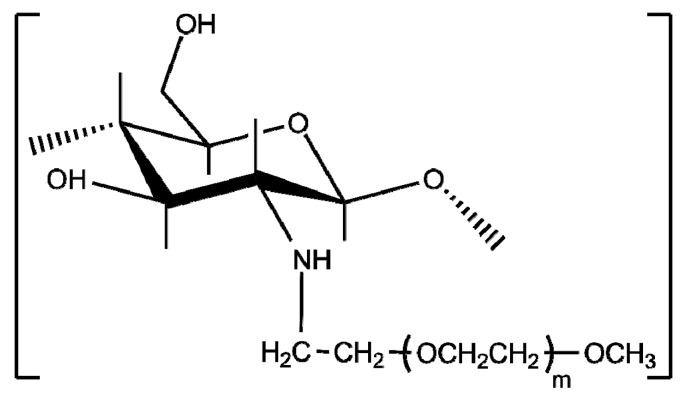
Chemical structure of chitosan-*g*-poly(ethylene glycol).

The grafting of poly(ethylene glycol) (PEG) onto chitosan backbone has been accomplished via PEGylation of amino groups throughout conjugation with methoxy PEG-nitrophenyl carbonate [131], methoxy PEG-succinimidyl carbonate [133], amidation with carboxylated PEG [134], or reductive amination (Figure 9) [129,130,132,135,136]. The use of “click chemistry” tools has also been reported for the *N*- [53,54] or *O*-PEGylation of chitosan [55,56]. However, the grafting onto the -OH groups at C-6 of chitosan structure is an alternative option of chitosan modification, because it allows the total availability of free amino groups. In this sense, some studies related to *O*-substitution graft copolymers have been developed by etherification reaction [24,128,137,138]. For this purpose, the amino groups of chitosan were protected with phthalic anhydride by the above-mentioned procedure. The resultant material (degree of substitution about 15%) shows solubility in a wide range of pH [128]. PEGylated chitosan has been considered as a bioactive delivery carrier for insulin [138], DNA [131], heparin [133], and albumin [135], among others. A detailed review of the methods of synthesis, characterization, and pharmaceutical applications of PEGylated chitosan derivatives could be consulted [155].

**Figure 10:**
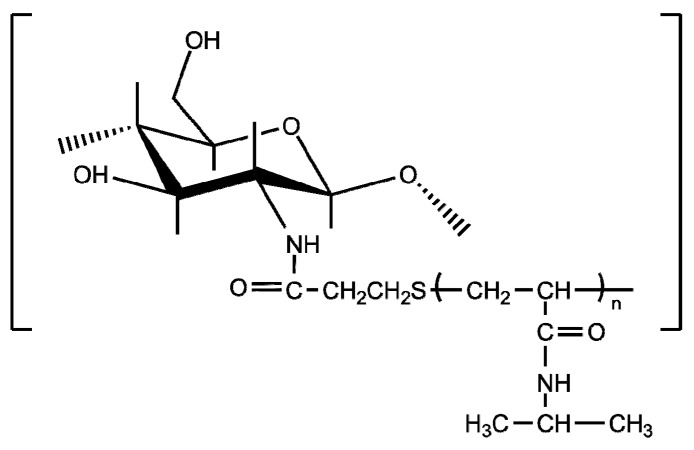
Chemical structure of chitosan-*g*-poly(*N*-isopropyl acrylamide).

Chitosan-*g*-PNIPAm copolymer has also been synthesized by the *graft onto* method via the amidation between carboxylic-end PNIPAm chains and chitosan amino groups using carbodiimide compounds like DCC [142], or EDC (Figure 10) [143,144,145]. Similarly, the same reaction, but between *O*-carboxymethyl chitosan and amino-end PNIPAm chains, has also been proposed [146], having the advantage of leaving the amino groups free. Bao et al. have also made use of “click chemistry” reactions to anchor PNIPAm chains onto chitosan backbone [57,58]. Due to its thermoresponsive behavior, this copolymer forms hydrogels in situ, which favors some properties as enhancement of drug residential time, ocular absorption, pharmacokinetics, and bioavailability of hydrophobic drugs [13,142,145].

**Figure 11:**
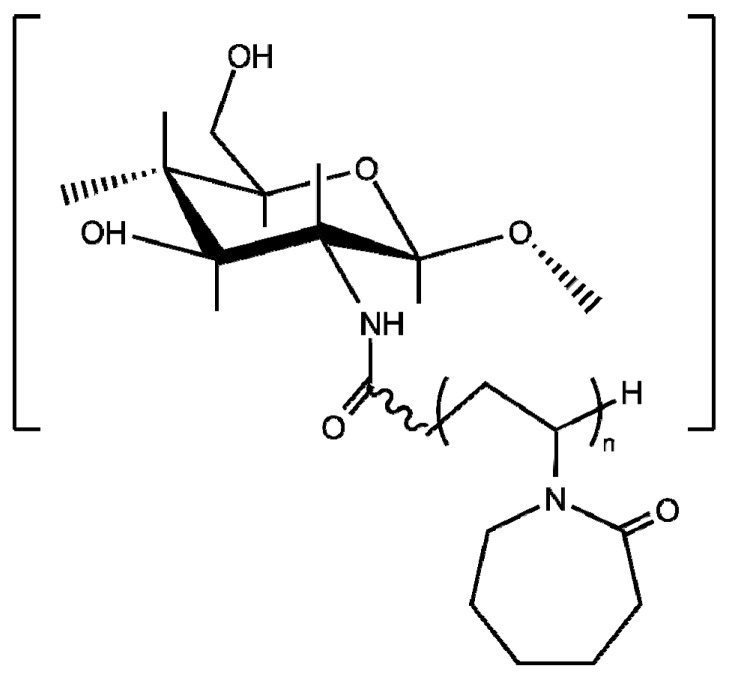
Chemical structure of chitosan-*g*-poly(*N*-vinyl caprolactam).

The *grafting onto* approach to synthesize chitosan-*graft*-PVCL has been conducted by the amidation between PVCL-COOH and chitosan amino groups using EDC/NHS system [147,148,149,150,152,153] or DMTMM (Figure 11) [151,154].

**Figure 12:**
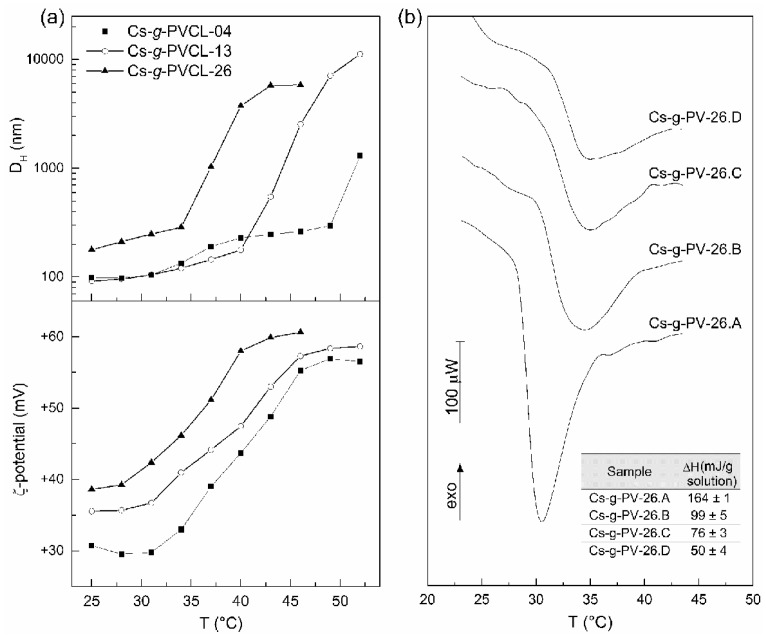
(**a**) Dependence of the hydrodynamic diameter, D_H_, on temperature of chitosan-*g*-PVCL aqueous solutions (pH 6) for different number-average molecular weights of PVCL-grafted chains (4, 13, and 26 kDa). Reprinted by permission from Springer Nature: [154], Copyright 2015. (**b**) Micro-DSC curve of 10 wt % aqueous solutions (pH 6) of chitosan-*g*-PVCL, varying the spacing between grafted side chains. Reprinted from [151], Copyright 2015, with permission from Elsevier.

It has been established that the molecular architecture of this copolymer plays a prominent role in their thermoresponsive properties (LCST within 34–45 °C) [151,154]. Figure 11 shows the dependence of the phase transition on the length of the grafted chain or the closeness between them along the chitosan backbone. The increment of the length of the grafted chains implies that longer hydrophobic segments appear, which favors polymer-polymer long-range interactions and as a result, a lowering of the phase transition temperature takes place (Figure 12a). The spacing between PVCL chains along the chitosan backbone also affects the transition: as they are closer, the lower the cloud point temperature and the greater the enthalpic change (Figure 12b). As the spacing between grafted chains is more reduced, the hydrophobic intercatenary interactions between PVCL segments are favored, giving rise to the above-mentioned behavior [151]. Indulekha et al. reported the study of the chitosan-*g*-PVCL gel as a transdermal drug delivery system for pain management, which showed biocompatibility and drug permeation through in vitro skin test [153]. Jayakumar et al. have studied chitosan-*g*-PVCL-based nanoparticles as a promising candidate for cancer drug delivery [147,148,149,150,152].

Table 1 presents a compendium of the most representative monomers that have been grafted into chitosan, as well as the main applications proposed.

### 3.4. Chitosan Network Systems Prepared by Radiation

Ionizing radiation constitutes an environmentally friendly tool for preparing graft copolymers from chitin and chitosan. Fundamentally, UV- and γ-radiation have been used for the preparation of chitosan derivatives. UV-initiated polymerization has some benefits, such as lower reaction temperature, fewer amounts of initiator, higher reaction rate, and shorter polymerization times, among others. The principal disadvantage of this method of modification is the absence of specificity. Usually, the resultant radiation-based graft copolymers tend to exhibit a crosslinked network structure.

On the one hand, chitosan-based graft copolymers have received special attention for applications as flocculants due to their biodegradability, absorption, and charge neutralization ability, among others. Some of those materials are based on acrylic monomers [156,157,158,159,160] and display important flocculation properties. There are also reports of radiation-induced chitosan grafted with poly(maleic acid) showing high sorption capacity of Co(II) [161,162]. On the other hand, Burillo et al. have developed thermosensitive graft copolymers based on chitosan derivatives by gamma radiation [163,164,165,166]. 

## 4. Chitosan-*grafted*-Cyclodextrin Derivatives

**Figure 13:**
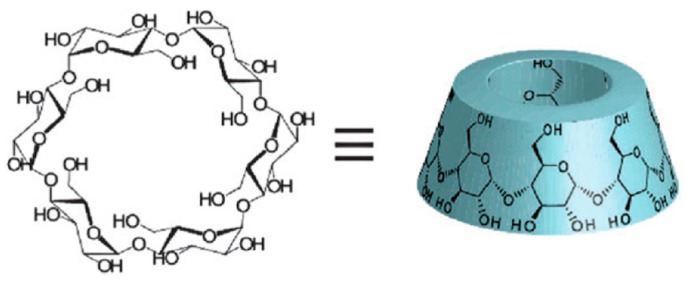
Structure of α-cyclodextrin (formed by six glucosidic units). The arrangement of the external hydrophilic surface and the relatively hydrophobic internal cavity is evident. Reproduced from [173] with permission of The Royal Society of Chemistry.

**Figure 14:**
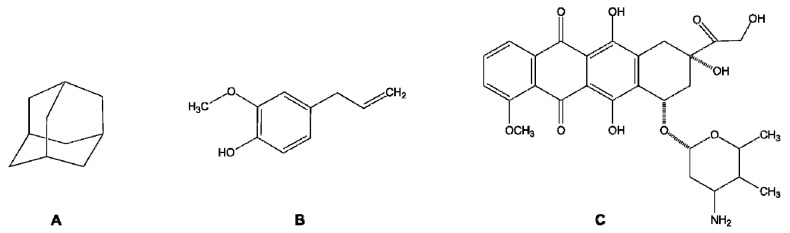
Chemical structure of (**A**) adamantane, (**B**) eugenol, and (**C**) doxorubicin.

Supramolecular polymer chemistry has gained interest in macromolecular research. A number of molecular architectures have been introduced to develop new materials, in which cyclodextrins (CDs) have been extensively used. CDs are non-toxic cyclic oligosaccharides, formed by 6 to 9 α-d-glucose units linked by (1→4) glycosidic bonds (Figure 13). They possess a truncated cone-shape geometry, with a hydrophilic external surface and a relatively more hydrophobic internal cavity [167]. This arrangement favors host-guest interactions through the inclusion of a wide variety of small organic molecules (which aremainly hydrophobic) such as adamantane, eugenol, doxorubicin, etc. (Figure 14) [168,169,170]. This important property makes CDs an effective molecular carrier during the design of advanced drug delivery systems. According to Rekharsky and Inoue, the general tendencies of the dependence on thermodynamic quantities can be understood in terms of hydrophobicity, steric effects during the guest-host interaction, the involved guest-host hydrogen bonding, and the flexibility of the guest molecule [171]. Thermodynamic studies about the stability of the inclusion complex demonstrated that the enthalpy gain due to the guest inclusion is compensated for by the loss of entropy that results from the considerable conformational changes that take place in the CDs during the complexation and the entropy gain due to the desolvation of both host and guest [171,172].

Many investigations have been carried out with the aim of proposing methods to prepare chitosan grafted CDs derivatives in order to take advantage of both the mucoadhesive properties and reactive functional groups of chitosan and the ability of CDs to interact with hydrophobic guest substances [174,175,176]. In the presence of a guest molecule, chitosan-*g*-CDs solutions could form intramolecular and intermolecular complexes, which can lead to a large increase in the viscosity or to the formation of temporary and reversible supramolecular network systems. Consequently, an adequate control of the grafting reaction is of utmost importance for the regulation of molecular architecture, and therefore the behavior and properties of the polymer materials. For this purpose, the methods available for chemical modification of chitosan can also be used to graft cyclodextrin. So far, the following main procedures have been proposed:

1. Reductive amination reaction. Usually, the CD is modified in order to attach an aldehyde group. The inclusion of the CD moieties into the chitosan backbone is carried out by the formation of a Schiff base, followed by the reduction with a proper agent. The reductive amination procedure is one of the most used, because it is a simple, easy, and slightly degradative method [169,177,178,179].
2. The second most important method is via amidation of CDs modified with a carboxylic group with the amino groups of chitosan. In this case, two strategies have been applied: (i) the classic condensation reaction [180,181], and (ii) by amidation using coupling activators of the carboxylic acid group, like EDC/NHS [175,182,183,184,185,186]. The former reaction requires high temperatures due to the high activation energies involved, while the use of condensation agents in the later selectively promotes the formation of the amide bond in aqueous solution under mild and controllable conditions.
3. The nucleophilic substitution of halides or tosyl groups by chitosan amino groups is another recurrent way to attach CDs into the chitosan backbone [168,170,187,188,189,190,191,192].
4. A method so far little used but which, in the future, can provide derivatives with a high regioselectivity, is anchoring β-cyclodextrin onto chitosan by click chemistry. In this way, using the Huisgen cycloaddition reaction, β-CD chains have been grafted onto the chitosan backbone through the amino group (position 2) [59] or to the O-6 [31].
5. Other methods, among which (i) the preparation of epoxy-activated chitosan and its reaction with hydroxyl groups of CD [193] or (ii) the anchoring of CD into chitosan using 1,6-hexamethylene diisocyanate [194,195,196], among others, can be mentioned.

In this sense, Auzély-Velty and Rinaudo have reported a procedure in which a monosubstituted β-CD, possessing a d-galacturonic acid group on the primary face of CD, was grafted onto the chitosan backbone by reductive amination reaction [169,178]. The characterization of the graft copolymer confirmed a successful inclusion of CD on the chitosan chain with almost no degradation of the polymer. These authors also observed a slight reduction in the solubility of the derivative (at grafting degrees 10–12%) as compared with that of the pristine chitosan [178]. At a given concentration, the viscosity of the copolymer solution is higher than that the original chitosan, confirming the presence of interchain interactions induced by the presence of grafted CD [169].

The host properties of CD and chitosan-*g*-CD were comparatively studied toward a low or high molecular weight guest. In the former case, 4-tert-butylbenzoic acid and (+)-catechin low molecular weight guests were chosen, and the inclusion complex was analyzed by means of NMR [178]. Experimental data corroborated that the complexation of 4-tert-butylbenzoic acid is a dynamic process, in the sense that the guest molecule is constantly switching between the free and bound states. Moreover, it was possible to conclude that chitosan-*g*-CD exhibits the same host-guest properties as the native CD toward the low molecular weight hydrophobic guest, suggesting that the grafting process does not have a significant influence over the binding capacity of CD [178]. In the second case, the interaction of chitosan-*g*-CD with two macromolecular guests (adamantane attached to chitosan or poly(ethylene glycol)) was evaluated [169]. On the one hand, NMR analyses demonstrated that the hydrophobic sites of the macromolecular guest interact with the grafted CD moieties in the same way as with the non-grafted one. On the other hand, rheological experiments showed that PEG end-capped with adamantane mixed with CD-chitosan solutions promote a significant increase in the viscosity due to cross-linking of CD-chitosan chains through host-guest inclusion complexation with PEG-di-adamantane guest. Nevertheless, when the complexation takes place with the chitosan-di-adamantane derivative, a gel-like behavior is appreciated [169]. These characteristics of the inclusion complex with di-adamantane macromolecular derivatives open interesting possibilities to produce advanced materials with controlled sol-gel properties.

**Figure 15:**
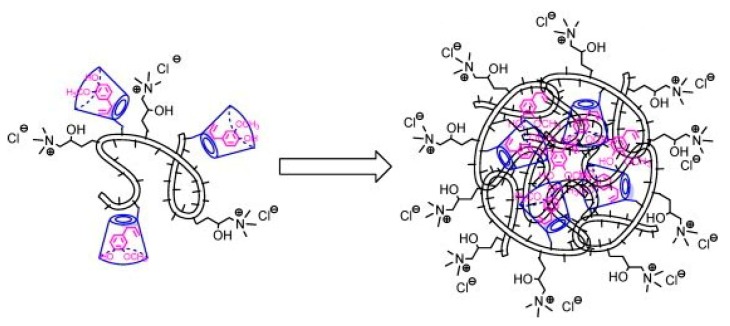
Schematic structure of inclusion complex between eugenol (pink) and quaternized chitosan (black) grafted with β-cyclodextrin (blue) forming self-aggregated micellar structures. Reprinted from reference [168], Copyright 2012, with permission from Elsevier.

One of the drawbacks of chitosan-*g*-CDs as a drug delivery system is the poor solubility of chitosan at neutral pH values. In this context, Sajomsang et al. have proposed the quaternization of chitosan amino groups in order to obtain a water-soluble grafted biopolymer [189,190]. Synthesis strategy involves the quaternization of previously prepared chitosan-*g*-CDs, carried out by the nucleophilic substitution of the remained free amino groups, yielding a water-soluble quaternized chitosan-*g*-CD. The degree of quaternization (DQ) reached values between 60 and 85%. The mucoadhesive properties of the grafted polymer were dependent on the DQ being stronger as the DQ increases, while its cytotoxicity does not show any dependence with the DQ [190]. The formation of an inclusion complex between the quaternized chitosan-*g*-CDs and eugenol as model guest molecule has also been studied. In this case, it was confirmed that eugenol was included in the hydrophobic cavity of CDs, but a self-aggregated micelle-type structure was formed, within which extra eugenol molecules were entrapped as illustrated in Figure 15. The greatest mucoadhesion was attained with the complex having 11% CD substitution, which suggests that in this case, electrostatic interaction has a key role in governing the adhesion between mucin and the chitosan derivative [168]. Moreover, an enhanced mucoadhesion was reported for this system when CDs were attached to the chitosan backbone throughout a citric acid molecule. This effect is possibly due to additional intermolecular hydrogen bonding between the carboxyl and hydroxyl groups from the citric acid spacer and mucus glycoprotein [181,197].

Another extensive coupling method used to graft CD into chitosan chain is based on amidation reaction. This reaction occurs among a component containing a free amino group, like chitosan, with a substituted carboxylic acid-cyclodextrin to generate the amide bond. This reaction is mediated by diimide derivatives; among them, EDC is the most used due to its water solubility. Daimon et al. described the preparation of a chitosan-*g*-CDs by the condensation reaction of chitosan and β-CD-carboxylate [175,176]. The interaction between chitosan-*g*-CDs and insulin was evaluated. Insulin was strongly bound to β-CD residues due to the specific host-guest inclusion complex with insulin. The electrostatic interactions between chitosan-*g*-CDs and insulin allowed a strong binding in a wide range of pH [175]. The conclusion of several studies is that chitosan-*g*-CDs have the remarkable potential to be applied in the delivery of peptides and proteins as an efficient delivery carrier [175,176,186]. 

Kono et al. described the preparation of a hydrogel based on carboxymethyl chitosan and carboxymethyl CD. A reductive amidation reaction was conducted employing EDC-NHS as the coupling agent. It allowed simultaneous grafting of CD onto chitosan and crosslinking. Acetylsalicylic acid was chosen as a model drug to explore its properties as a carrier for drug delivery system. According to their results, the observed drug release profile could be attributed to the formation of an inclusion complex of aspirin inside CD cavity [183].

Apart from the aforementioned applications for controlled release systems, other studies that aimed at the use of chitosan/cyclodextrin materials for the removal of metals or organic micropollutants from wastewaters have been described as well [198,199]. For example, Zhao et al. prepared chitosan- β-cyclodextrin absorbent material using EDTA as cross-linker. According to these authors, “chitosan chain is considered as the backbone, and the immobilized cyclodextrin cavities capture the organic compounds via host-guest inclusion complexation, while EDTA-groups complex metals” [199]. A β-cyclodextrin-chitosan-graphene oxide composite material has also been proposed. It is claimed that this material is appropriate for the removal of manganese ions [198].

Finally, it should be noted that there is an increasing number of publications in which chitosan and cyclodextrin are used as important components in the preparation of nano-vehicles or stimuli-sensitive carriers [170,185,191,200,201,202,203].

## 5. Dendronized Chitosan

Dendrimers are commonly represented as highly symmetrical molecules, displayed in tiers with an algorithmic growth. They are characterized by high-end functional groups located on the surface of a spherical conformation, which lead a molecule that owes a large number of functional sites that are easily accessible. This typical architecture influences the physical properties, like solution behavior, especially at high molecular weights. In dendrimer construction, two synthetic approaches have been employed: divergent and convergent. In the former, stepwise growing occurs from the center by means of a series of highly selective reactions over a single molecule, whereas in the latter, the synthesis begins in the periphery and ends in the core. Despite the important biomedical applications of dendrimers as viral and pathogenic cell adhesion inhibitors, references about dendronized chitosan derivatives are still scarce [204]. Here is a general brief description of these novel chitosan derivatives.

**Figure 16:**
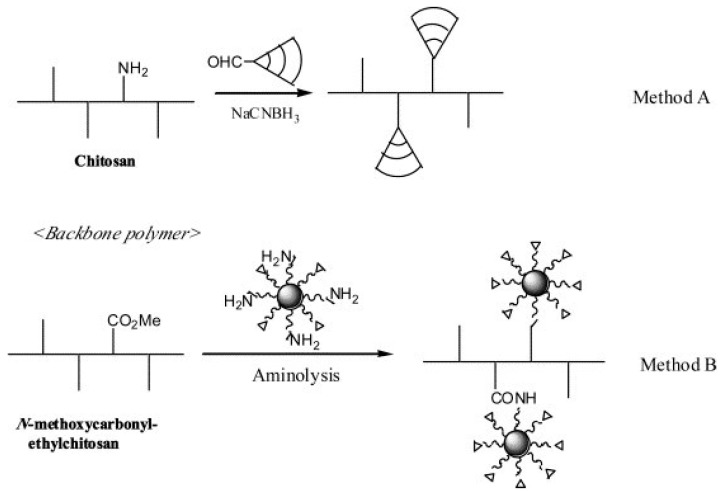
Synthetic strategy on chitosan–dendrimer hybrid. Reprinted from [12], Copyright 2004, with permission from Elsevier.

Some of the first reports of the preparation and characterization of chitosan dendrimers are those presented by Sashiwa et al. [205,206,207,208]. They reported the preparation of sialic acid-bound dendronized chitosan using gallic acid as the focal point and tri(ethylene glycol) as spacer arm. It was suggested to be a non-toxic alternative and an inhibitor of hemagglutination of influenza viruses. Sashiwa and Aiba have proposed two main strategies for the synthesis of chitosan dendrimers (Figure 16): in *Method A,* dendrimers bearing an aldehyde group are previously synthesized, and then reacted with chitosan amino groups by reductive *N*-alkylation; while *Method B* is based on the binding of chitosan to the dendrimer surface, allowing the use of available amino-dendrimers [12].

A bioabsorbent for heavy metals, composed of different generations of poly(amido amine) (PAMAM) dendrimers, was achieved by divergent approach synthesis. The addition of methyl acrylate over amino groups of chitosan powder surface was driven by the Michael addition reaction followed by the amidation of terminal groups with ethylenediamine. Different generations of PAMAM were obtained by the subsequent propagation of PAMAM. Results indicate that materials with higher generations of dendrimer exhibit greater Pb^2+^ adsorption capacity [209].

**Figure 17:**
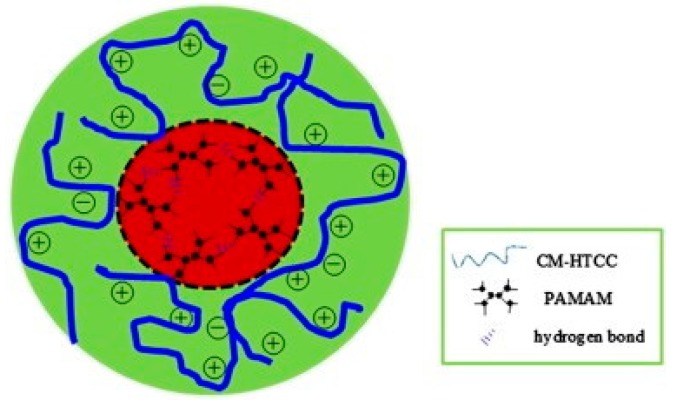
Putative schematic structure of CM-HTCC/PAMAM dendrimer core-shell nanoparticles. Reprinted from [210], Copyright 2012, with permission from Elsevier.

The preparation of a water-soluble *O*-carboxymethyl *N*-[(2-hydroxy-3-trimethylammonium) propyl] chitosan (CM-HTCC)/PAMAM dendrimer has also been described [210,211]. These nanoparticles are composed of a PAMAM core dendrimer and an outer CM-HTCC shell attached to it (core-shell nanoparticles), as can be appreciated in Figure 17. The synthesis of this dendronized chitosan involves a two-step reaction: the activation of carboxylic groups in CM-HTCC and the subsequent condensation reaction. The obtained chitosan dendrimer could self-aggregate into core-shell nanoparticles due to the combination of hydrophobic and electrostatic interactions and hydrogen bonding. These dendrimer nanoparticles exhibited antibacterial activity against Gramm negative bacteria as *E. coli*.

Similar nanostructures were also prepared with magnetite nanoparticles and dendritic branches with carboxymethyl chitosan terminal groups [212]. These dendrimers exhibit selective adsorption for anionic and cationic compounds at specific pH, and their potential use to remove dyes was successfully proved.

## 6. Chitosan Modification Using Ionic Liquids

The ionic liquids (IL) have become a versatile media in which to perform chitin and chitosan derivatization that was not available few of decades ago. Ionic liquids are salts that remain liquid below 100 °C, in a practical sense, are those salts that should be handled as liquids at room temperature. Most of them are formed by uneven ionic moieties, usually large cations paired with anions of relatively smaller size. The combination and modification of cations and anions make it possible to obtain ionic liquids with diverse chemical characteristics and functional properties. Thus, IL have been praised as customizable solvents, some of them with remarkable properties that have found their way into industrial scale applications. Many IL have been also classified as “green” solvents due to their reduced vapor pressure, conventional non-flammability, and exceptional solvation potential [213,214].

**Figure 18:**
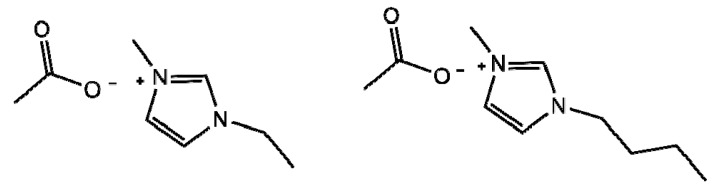
Chemical structure of the acetate salts of 1-ethyl-3-methylimidazolium, Emim, and 1-butyl-3-methylimidazolium, Bmim.

IL’s capacity to dissolve polysaccharides was first reported in 1934. However, this does has not received considerable scientific attention, until recently. One of the main focuses of interest has been the capacity of some IL to dissolve typically intractable polysaccharides as cellulose or chitin [215,216,217,218]. Imidazolium-based IL, particularly 1-ethyl-3-methylimidazolium (Emim) and 1-butyl-3-methylimidazolium (Bmim) in chloride or acetate form (Figure 18), is commonly used to prepare chitin and chitosan solutions that could reach relatively high concentration (over 10 w%). Other types of IL have been reported to dissolve chitosan to different extents, for example, pyridinium-based IL functionalized with sulfonic acid [219] or amino acid-based IL [220]. The chitosan-IL solutions provide alternative media to get homogeneous reaction conditions and also enable derivatizations that are not favored in aqueous environments. The availability of this type of chitin-chitosan solvent system began to gain relevance in scientific research and applications development.

**Figure 19:**
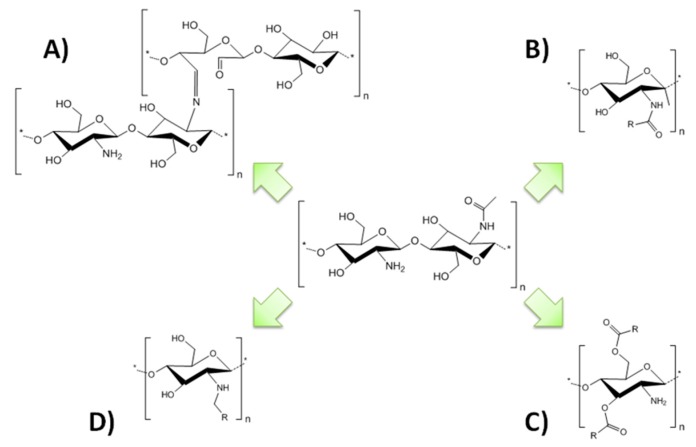
Some examples of chitosan derivatization made in IL. (**A**) Chitosan-graft-oxicellulose, (**B**) *N*-acylation, (**C**) *O*-acylation, and (**D**) Alkylation.

Actually, several types of chemical modifications of chitin-chitosan in IL have been reported. Some of them have been compiled in focused reviews [221,222]. Chitosan has several functional chemical groups that are susceptible to react, which allow the production of a range of derivatives and grafting. Below is a succinct summary of the most relevant chitosan derivation procedures in IL reported in the literature, and some examples of the obtained products are included in Figure 19.

### 6.1. Acylation

Acetylation was one of the first chemical modification procedures performed on chitin-chitosan dissolved in ionic liquids. Homogeneous acetylation of chitin and chitosan in halide imidazolium-based IL has been reported [223,224]. Based on the degrees of substitution and spectroscopic evidence reported, both *N*-acetylation and *O*-acetylation was achieved indistinctly. With IL, the acetylation of chitosan proceeds in mild and homogeneous conditions, making this methodology more straightforward compared to usual procedures [222]. Other acylation procedures have been reported. The IL Bmim acetate (BmimAc) was used as the reaction solvent to obtain *N*-linoleyl chitosan oligomers. Narrow-distribution low molecular chitosan was used as starting material that was acylated with linoleic acid using EDC and 4-(dimethylamino) pyridine (DMAP) as catalysts under mild reaction conditions. The nanomicelles of the obtained amphiphilic molecules are proposed as drug vector [225]. Similarly, the use of glycine chloride ([Gly]Cl) aqueous solution as media to synthesize *N*-acyl chitosan derivatives (i.e., *N*-maleyl, *N*-succinyl chitosan, and *N*-acetylated) was reported as a procedure for obtaining fibers with improved mechanical properties [226]. Another acylation type modification was achieved by reacting chitosan with monomethyl fumaric acid mediated by EDC. The reaction media was an aqueous solvent system that included 4 w% of the IL, 1-sulfobutyl-3-methylimidazolium trifluoromethanesulfonate (BSmimCF_3_SO_3_). The product, monomethyl fumaric-chitosan amide, has improved water solubility and antioxidant activity [227]. Chitosan has been also reacted with a carboxyl group-bearing IL (1-carboxypropyl-3-methyl imidazolium chloride) to obtain an acyl conjugate. Spectroscopic techniques (NMR and FTIR) were used to elucidate the structure of the chitosan-ionic liquid conjugate. This compound shows good anion adsorption performance and was proposed for wastewater treatment [228].

### 6.2. Alkylation

Several alkylation-type modifications of chitosan have been done using IL as media and catalyst. The nucleophilic substitution of 2,3-epoxypropyltrimethyl ammonium chloride (EPTAC) onto chitosan, using ionic liquid of 1-allyl-3-methylimidazole chloride (AmimCl) as a homogeneous reaction media, produced *N*-[(2-hydroxyl)-propyl-3-trimethyl ammonium] chitosan chloride (HTCC). In this system, the attack of the amino groups of chitosan on the C atom with less steric hindrance in EPTAC is thermodynamically favored, according to quantum chemistry calculations [229]. Chitosan was reacted with four alkyl halides in a basic form of the Bmim IL to prepare a series of alkylated chitosans with different carbon chain substituents (i.e., ethyl-, butyl-, dodecyl-, and cetyl-chitosan). The analysis of FTIR spectra indicates the occurrence of *O*-alkylation; however, the *N*-alkylation prevails at the reaction conditions used. The antibacterial activity of alkylated chitosans decreased with the growth of the DS or the growth of the carbon chain [230]. Another report of *N*-alkylation of chitosan in IL is the production of HTCC in AmimCl [231]. In contrast, there are few examples of *O*-alkylation of chitosan achieved in IL. Dodecanol was selectively linked to hydroxyl groups of chitosan using *N*,*N*′-carbonyldiimidazole as a bonding agent and BmimCl as homogeneous media. The authors attribute the selective alkylation of hydroxyl groups of chitosan, without protecting amino groups, to the particular properties of the ionic liquid solvent [232].

### 6.3. Grafting

The solvent capacity of several IL has been used to achieve grafting on chitin or chitosan. Chitin graft polystyrene was obtained by atom-transfer radical polymerization (ATRP) in AmimBr [233]. Methacryloyloxyethyl trimethylammonium brushes were formed on chitosan by single electron transfer living radical polymerization in BmimCl [234]. The synthesis of chitosan graft polyethylenimine copolymers was developed in BmimAc [235]. Two different research groups have reported the chitosan grafting with polycaprolactone using IL as a solvent. Wang and collaborators used EmimCl as solvent and stannous octoate as catalyst [236], whereas Yang and co-workers used a ring-opening graft polymerization route with *N*-protected chitosan dissolved in BmimAc [229]

Ionic liquids allow the homogeneous mixture of polysaccharides in solution. This has been used to produce several composite materials. Furthermore, these solvent systems have enabled the possibility of carrying out inter-polysaccharide reactions that have been proven to be difficult to do in other media. Thus, it was possible to produce chitosan graft oxycellulose using a mixture of two IL, AmimCl as the solvent, and 1-sulfobutyl-3-methylimidazolium hydrogen sulfate (SmimHSO_4_) IL as the catalyst of the reaction [237]. Another example is the covalent linking of chitosan and xylan through the Maillard reaction in BmimCl [238].

### 6.4. Other Derivatizations

The crosslinking of chitosan in IL has been explored. Chemical ionogels were obtained crosslinking chitosan with glutaraldehyde in EmimAc [239]. Recently, the design of a dicationic IL (1,10-(butane-1,4-diyl)bis(3-(4-bromobutyl)-1H-imidazole-3-ium)bromide) used as crosslinking agent for chitosan was reported. The composite materials of chitosan crosslinked with IL were tested as catalysts of the cycloaddition reaction of CO_2_ with various epoxides [240]. 

Other derivatization reactions of chitosan performed in ionic liquids solutions include the formation of a Schiff base conjugate using BmimCl as solvent [241] and the sulfonation of chitosan in an aqueous solvent system containing [Gly]Cl [242].

### 6.5. Degradation

A homogeneous reaction media like that obtained using IL represents an opportunity window to test diverse modifications in the chemical structure of chitin and chitosan. One of the basic modifications of these polysaccharides is the deacetylation. This has been achieved by hydrothermal treatment using aqueous BmimAc as reaction medium and catalyst [243]. However, there are more scientific reports on the hydrolysis of chitin and chitosan in IL. 

A mixture of BmimCl, BmimBr, and hydrochloric acid was effectively used to depolymerize chitin [244]. Improved reaction rates were reported when chitosan dissolved in AmimCl was treated with sulfonic acid-functionalized ionic liquids based on propylpyridinium and microwave irradiation [219]. An aqueous solution-ionic liquid biphasic catalytic system was proposed for the oxidative degradation of chitosan. Chitosan was dissolved in diluted HCl and the hydrophobic ionic liquid 1-*N*-butyl-3-methylimidazolium bis((trifluoromethyl)sulfonyl) imide ([bmim][Tf_2_N]) containing with iron(II) phthalocyanine (FePc) complete the oxidative catalytic system [245]. Furthermore, a nitrogen-containing furan derivative has been obtained directly from chitin dissolved in a range of imidazolium-based IL, containing HCl or HBr as additives, after a thermal treatment [246].

### 6.6. Biocatalyzed Reactions

Ionic liquids have been also used as effective media for biocatalyzed reactions. It is considered that many enzymes, particularly those that tolerate conventional organic solvents, can achieve comparable activities in ionic liquids. Moreover, ionic liquid solvent systems could overcome some limitations that are observed in the biotransformation of highly polar substrates, such as polysaccharides [213]. Consequently, several research groups have studied enzymatic modifications of chitin-chitosan using IL as reaction media or additive. Bacterial and fungal chitinases dispersed in an aqueous solvent system containing EmimAc were applied to produce monomers and oligosaccharides from chitin. A notorious enzymatic activity reduction was observed when IL concentration was over 20 v% [247]. Chitosan oligomers were produced with amylose in a [Gly]BF_4_ aqueous medium. Similarly, an enzymatic activity reduction was observed when the IL concentration went over 8 v% [248]. On the other hand, commercial lipase was used for the synthesis of chitosan esters via transesterification with methyl palmitate. The reaction media contain a mixture of a hydrophilic IL, EmimAc, and a hydrophobic IL, Bmim tetrafluoroborate [249].

The ionic liquids have become a promising solvent platform for controlled chemical modification of chitosan. There are examples of controlled reactions, even regioselective, derivatization of chitosan using IL as media, additives, or catalysts. The “customization” of IL could provide tunable homogeneous phase media to circumvent the common drawbacks of heterogeneous conditions (i.e., require harsh reaction settings, high variability, low product yields, extended reaction times, etc.) [221,222]. Most of the cited authors in this section remark the “green” solvent condition of IL referred to their low vapor pressure, non-flammability, and thermal and chemical stability. Furthermore, the reuse and recycling of IL has received particular attention. After reaction, the chitosan derivatives are usually recovered by precipitation using miscible non-solvents, e.g., alcohol-water mixtures or organic solvents, depending on the utilized IL and the obtained chitosan derivative. The residual solvents and washing liquids could be distilled to recover the IL. However, the main concerns about the use of IL focus on their biocompatibility and their cost, as they are not readily available yet. The application of IL for polysaccharide processing is relatively recent subject; the possibilities enabled are numerous; thus, considerable research effort is ongoing worldwide.

## 7. Conclusions

In this review, we summarize and discuss the latest advances in methods and strategies of chitosan functionalization, such as the click chemistry approach, *grafting onto* copolymerization, coupling with cyclodextrins, as well as reactions in ionic liquids. A better understanding of the close relationship between chemical structure and properties is an imperative condition for designing innovative materials. This involves synthesizing substances whose macromolecular architecture is controllable so that the required properties can be optimized and maximized. Throughout this article, several possibilities for combining the distinctive characteristics of chitosan with other interesting molecules, within a context that allows the governance of the architecture of the resulting derivatives, have been demonstrated. The materials produced could be used in exciting technological applications such as sensors, actuators, as a controllable membrane for separations, in the food industry, nanotechnology, and in biomedical and biotechnological fields including drug delivery and tissue engineering. The application of novel techniques of polymer modification with an adequate control of the regioselectivity has allowed important advances in recent years. We should expect that in the near future, new experimental techniques will appear, and materials with properties for specific applications will surely arise.

## Figures and Tables

**Table 1:** Main monomers used for derivatization of chitin and chitosan by grafting copolymerization.

Monomers	Applications	References
**Chitin “grafting from” copolymers**		
Acrylamide	Water absorbents, chelating agents	[82]
Acrylic acid	Water absorbents, chelating agents. Wound dressing. Nanofibers	[82,85,88]
Methyl methacrylate	Gel-like mass for biomedicine	[84]
Itaconic acid	Waste-water treatment	[86]
Indole	Antimicrobial activity	[87]
ε-caprolactone	Biomedical field	[90]
Glycidytrimethylammonium chloride	Wound healing	[91]
Pyrrole	Electrically-conducting material	[94]
**Chitosan “grafting from” copolymers**		
Acrylic acid	Controlled release devices, ion-exchange bioseparation, antibacterial activity, removal of heavy metal ions	[95,103,104,111]
*N*-butyl acrylate	Biodegradable packaging materials, recovery of heavy metals from waste waters	[105,109]
Iodine	Cervical antibacterial biomembrane	[110]
acrylamide-co-acrylic acid	Drug release hydrogels	[112]
Styrene	Recovery of heavy metals from waste waters	[105]
Aniline	Antibacterial activity	[121]
PNIPAm	Biomedical field: tissue engineering, drug delivery systems.	[96,97,98,99,100,101,102,106,107,108,113,123,145]
Lactide	Gene delivery, complex with DNA	[119]
ε-caprolactone	Nanoparticle stabilizer, drug delivery systems	[37,116,117,118]
*N*-vinyl-2-pyrrolidone	Antimicrobial activity, nanoparticle stabilizer	[115,116]
Carbamate (urethane)	Drug delivery systems	[120]
Indole	Antimicrobial activity	[87]
**Chitosan “grafting onto” copolymers**		
Pluronic	Injectable cell delivery carrier, gene expression, controlled release	[140,141]
ε-caprolactone	Drug carriers, antimicrobial activity	[125,126,127]
Ethylene glycol	Bioactive molecules delivery, polymeric surfactants, gene delivery, apoptosis-inducing activity.	[128,131,133,135,137,138]
PNIPAm	Drug/gene delivery,	[57,58,98,142,143,144,145,146]
PVCL	Controlled drug delivery systems	[147,148,149,150,151,152,153,154]
